# An Assessment of Magnitudes and Patterns of Socioeconomic Inequalities across Various Health Problems: A Large National Cross-Sectional Survey in Korea

**DOI:** 10.3390/ijerph15122868

**Published:** 2018-12-14

**Authors:** Ji-Yeon Shin, Jiseun Lim, Myung Ki, Yeong-Jun Song, Heeran Chun, Dongjin Kim

**Affiliations:** 1 Department of Preventive Medicine, School of Medicine, Kyungpook National University, Daegu 41944, Korea; jyshin@knu.ac.kr; 2 Department of Preventive Medicine, College of Medicine, Eulji University, Daejeon 34824, Korea; jslim@eulji.ac.kr; 3 Department of Preventive Medicine, College of Medicine, Korea University, 73 Inchon-ro, Seongbuk-gu, Seoul 02841, Korea; 4 Jeonnam communicable disease management support team, Chonnam national university Hwasun, Hwasun 58128, Korea; syjace@nate.com; 5 Department of Health Administration, Jungwon University, Goesan 28024, Korea; heeranchun@gmail.com; 6 Health Care Policy Research Department, Korea Institute for Health and Social Affairs, Sejong 30066, Korea; djkim@kihasa.re.kr

**Keywords:** socioeconomic inequalities in common health problems, socioeconomic factors, health inequalities in non-communicable diseases, health inequalities in mental diseases, health inequalities policy, Korea

## Abstract

Magnitudes of health inequalities present consequences of socioeconomic impact on each health problem. To provide knowledge on the size of health problems in terms of socioeconomic burden, we examined the magnitudes and patterns of health inequalities across 12 health problems. A total of 17,292 participants older than 30 years were drawn from the Korea National Health and Nutrition Examination Survey (KNHANES, 2010–2012). The age-adjusted prevalence ratios were compared across socioeconomic positions (SEPs) based on income, education, and occupation. The magnitudes of socioeconomic inequalities varied across 12 health problems and, in general, the patterns of socioeconomic inequalities were similar among groups of health problems (i.e., non-communicable diseases (NCDs), mental health, and subjective health states). Significant health inequalities across NCDs, such as diabetes, hypertension, ischemic heart disease, and arthritis, were observed mainly in women. Socioeconomic inequalities in mental health problems, such as depression, suicidal ideation, and suicide attempts, were profound for both genders and across SEP measures. Significant socioeconomic inequalities were also observed for subjective health. No or weak associations were observed for injury and HBV infection. The patterns of socioeconomic inequalities were similar among groups of health problems. Mental illnesses appeared to require prioritization of socioeconomic approaches for improvement in terms of absolute prevalence and relative socioeconomic distribution.

## 1. Introduction

Despite increased recognition, research on health inequalities, which reflect the distribution of a health problem across socioeconomic groups, has not been given high priority for options to guide health policy. One way to enhance the relevance of policies on health inequalities would be an examination of variation in health inequalities across health problems by providing knowledge on the burden of health problems particularly among populations with lower socioeconomic position (SEP). Evidence suggests that failure to reduce the prevalence of some types of diseases requires more selective focus on groups with lower SEP [1,2,3]. These groups are less responsive to public health interventions than groups with higher SEP and, in these cases, the prevalence of the condition is by far reflective of socioeconomic inequalities. Therefore, appraisal of the magnitude of health inequalities across multiple health problems may help clarify which conditions should be targeted for further reduction of the overall prevalence [4].

The magnitude of socioeconomic association varies across health problems and no single health problem wholly represents the variation in health inequalities [4,5]. To illustrate, socioeconomic inequalities are mostly presented for non-communicable diseases (NCDs) (e.g., diabetes, hypertension, ischemic heart disease, and arthritis) [5,6,7], but evidence is conflicting for cancer, communicable diseases, asthma, and headache/migraine [5,8,9], and no or reversed socioeconomic inequalities for allergy, skin disease, and breast cancer have been reported [5,9]. Among NCDs, socioeconomic inequalities for cardiovascular diseases are more obvious in Western countries [10,11], while the degrees of inequalities in Asian and developing countries [12,13,14] are less consistent. Furthermore, health inequalities may be diverse depending on the context of a country-specific health care system; in countries such as South Korea (hereafter, Korea), where the national health insurance coverage is relatively limited, out-of-pocket medical spending is the largest among the Organization for Economic Co-operation and Development (OECD) countries [15] and benefit coverage is heterogeneous across health problems. This suggests that socioeconomic stress on patients may be more affected by types of health problems in Korea.

Studies on health inequalities to date have included individual or limited numbers of health problems and comparability issues have often been a barrier to developing an overview of the magnitude of health inequalities across different health problems [7]. Considerable heterogeneity across studies in methodological settings (e.g., differences in socioeconomic measures, health indicators, and country-level contexts) hinder comparisons. Comparisons with comprehensive inclusion of an illness spectrum have mostly been related to mortality [16,17] or non-specific morbidity (e.g., self-rated health and long-term disabilities) [4,6] and research on specific morbidities have been scarce [5,18,19]. To avoid this limitation, the current study was conducted with a range of socioeconomic measures and health problems within a single dataset. Based on a large nationally-representative dataset, we examined examining whether the magnitudes of socioeconomic inequalities vary by health problems, whether the patterns differed by men and women and whether there are corresponding patterns of health inequalities among similar entities of health problems (e.g., NCDs, subjective health state, and mental health).

## 2. Materials and Methods

### 2.1. Data Source

We used data from the 2010–2012 Korea National Health and Nutrition Examination Survey (KNHANES), a nationally-representative survey of the non-institutionalized Korean population conducted by the Korea Centres for Disease Control and Prevention (KCDC). The survey uses a stratified multistage probability sampling design. For the KNHANES 2010–2012 dataset, 576 sampling units were randomly selected from the primary sampling units encompassing the target population in South Korea; households per sampling unit were selected for a total of 11,520 households. KNHANES has three components: a health interview, health examination, and nutritional survey. We used data from the health interview and health examination to obtain information on sociodemographic characteristics, medical history and laboratory measurements. Details of the survey have been described elsewhere [20]. Of the 25,534 individuals investigated in KNHANES 2010–2012, our study sample consisted of 17,292 subjects aged ≥ 30 years.

### 2.2. Measures

#### 2.2.1. Health Conditions and States

The KNHANES includes a large range of health conditions, which is used as Korean national statistics for disease prevalence. Among the listed health problems (Supplementary Table S1), based on expert consensus by three qualified medical doctors, we selected health conditions and states to represent wide range of the illness spectrum: (1) health conditions: NCDs, mental health, communicable disease, and injury; and (2) health states: self-rated health and quality of life. We excluded some conditions, when it is relatively mild condition among the same entities (e.g., anemia), when surveyed only for few years (e.g., osteoporosis), and when frequency is too rare to yield estimates (e.g., individual cancer). For NCDs, we assessed the prevalence of hypertension, diabetes, cancer, ischemic heart disease (IHD), and arthritis. For mental health, we assessed the self-reported depressive mood, suicidal ideation, and suicide attempts. As an example of a communicable disease and injury, we assessed the prevalence of hepatitis B and the one-year injury experience, respectively. For health states, we assessed the self-rated general health status and health-related quality of life using the EuroQol five-dimension questionnaire (EQ-5D). We considered a participant to have diabetes if they satisfied one or more of the following criteria; (1) fasting plasma glucose level ≥ 126 mg/dL, (2) currently taking anti-diabetic treatment (either insulin or oral anti-diabetic drugs), or (3) previous diagnosis of diabetes by a physician. Hypertension was defined, when one or more of following criteria was satisfied: (1) the average systolic blood pressure ≥140 mmHg, (2) the average diastolic blood pressure ≥90 mmHg, (3) taking antihypertensive medications, or (4) previous diagnosis of hypertension by a physician. Participants who had been diagnosed with angina pectoris or myocardial infarction by a physician were classified as having IHD. For arthritis, we limited subjects to those over 50 years old. We defined arthritis as a diagnosis of osteoarthritis or rheumatoid arthritis by a physician. Subjects were defined as cancer patients when they answered “yes” to the question “Have you ever been told by a physician that you have cancer or a malignancy of any kind?”

Self-rated health status was assessed using the question “Is your health in general excellent, good, fair, poor or bad?” The responses were grouped into binary categories: good (excellent, good or fair) and poor (poor or bad). The health-related quality of life was assessed using the EQ-5D. Participants were asked about their mobility, self-care, usual activities, pain/discomfort, and anxiety/depression. The response to each dimension was scored on one of three levels: no, moderate, or severe problems. A single EQ-5D utility index score was calculated using a specific Korean valuation set developed by the KCDC using a time trade-off protocol, which represents population-based preference weights for health states in Korea [21]. Scores on the EQ-5D index range from −0.171 to 1, and we divided the subjects into binary groups according to the median of EQ-5D scores to categorize the ‘better’ and ‘worse’ health-related quality of life group. Those who scored the median or better for EQ-5D scores were defined as being in the ‘better’ health-related quality of life group, while those with a score below the median were defined as being in the ‘worse’ health-related quality of life group. Subjects who answered “yes” to the following question were regarded as having a depressive mood: “Have you felt sad or desperate for ≥2 weeks such that it has affected your everyday life during the past year?”. Suicidal ideation was assessed by the question “In the past year, have you ever felt like dying?” If the subject answered “yes,” they were further asked about their suicide attempts with the question, “Did you attempt suicide in the past year?”

The prevalence of hepatitis B virus (HBV) infection was assessed according to the sero-prevalence of HBV surface antigen (HBsAg). HBsAg tests were performed using electrochemiluminescence immunoassays (E170, Roche, Mannheim, Germany) during the 2010–2012 KNHANES. HBsAg titres > 1 IU/mL were considered positive for HBV infection. Subjects who reported “yes” to the following question were regarded as having injury experience: “Have you ever had an accident or poisoning that you had to be treated for at a hospital or emergency room in the past year?”

#### 2.2.2. Socioeconomic Position Indicators

We used income level, educational attainment and occupational group as indicators of SEP. For income level, we used the equivalent household monthly income, calculated as monthly household income divided by square root of the number of persons in the household [22], and the population was divided into quartiles (low, mid-low, mid-high, and high) by year and gender. With regard to educational attainment, we evaluated the elderly and the middle-aged separately, as there is a gap in the average education level between these two age groups. For the middle-aged population (30–64 years), educational attainment was categorized into elementary school graduate and below, middle school or high school graduate, or college graduate and above. For those aged ≥65 years, educational attainment was categorized into elementary school graduate or below, middle school graduate, or high school graduate and above [13]. Analyses of occupations were conducted in the population aged 30–64 years, excluding soldiers, students, and homemakers. Occupations of the participants were classified into nine occupational groups (i.e., managers; professionals and related workers; clerks; service workers; sales workers; skilled agricultural, forestry, and fishery workers; craft and related trades workers; equipment, machine operating, and assembling workers; and elementary workers) according to the major categorizations of the sixth Korean Standard Classification of Occupations [23] based on the subjects’ answers. We classified the nine occupational groups into three categories: manual workers, service or sales workers, or managers and office workers.

### 2.3. Statistical Analysis

Simple descriptive statistics were used to evaluate the basic characteristics of the study population. Inequalities were measured using both absolute and relative measures. The age-adjusted prevalence difference (PD) and prevalence ratio (PR) between the lowest and highest SEP groups were used as absolute and relative inequalities indicators, respectively. The reference groups for the PRs were the highest income quartile, highest education level, and managers or office workers. The 95% confidence intervals of PR and PD were calculated by the z-distribution. In the case of PR, the confidence intervals were corrected for skewness using Gart and Nam’s Score [24]. We used the 2005 Korean census population as the standard population for direct age standardization to take into account the variation in age structure in each SEP hierarchy. In the results, we present the health inequalities using relative measures, as these more effectively show the health inequalities with large baseline differences in the prevalence of various health problems. Patterns of health inequalities across health problems were narratively assessed by comparing estimates obtained from separate analyses for each health problem. We also presented the PDs and PRs comparing all SEP categories in total participants, men and women separately in the Supplementary Tables (Supplementary Tables S1–S6).

All statistical analyses were performed using the software package SAS (ver. 9.4, SAS Institute, Cary, NC, USA). We incorporated the sampling weights of the KNHANES in all analyses.

## 3. Results

Among the 17,292 participants, 43.5% were men, and the proportions of middle-aged and older-age groups were similar between men and women. The proportion of low-income participants was significantly higher in women (23.1%) than in men (19.1%). Women were more likely to have lower educational attainment in both the middle-aged and older-age groups; e.g., 81.9% of women and 45.5% of men had a lower educational level among the elderly. Manual occupation was the most common in both genders, and the proportion of manual occupation was higher for men (51.0%) than women (41.4%). Among the NCDs, diabetes, and hypertension were more prevalent in men, and arthritis was far more prevalent in women, while the prevalence of cancer and IHD were similarly low in both genders. The prevalence of mental health problems (depressive mood and suicidal ideation, with the exception of suicide attempts) and poorer health states (self-rated health status and quality of life) were substantially higher in women than in men. The prevalence of hepatitis B and injury were similar between men and women (Table 1).

Overall, mental health showed the largest magnitudes of health inequalities in both male and female participants. For example, the PRs for depressive mood, suicidal ideation and suicide attempts between the highest and lowest income groups were 2.03 (95% confidence interval (CI): 1.47–2.78), 2.28 (95% CI: 1.70–3.06) and 6.43 (95% CI: 2.05–20.15) in men and 1.97 (95% CI: 1.59–2.44), 2.21 (95% CI: 1.77–2.75) and 6.47 (95% CI: 2.17–19.32) in women, respectively. Socioeconomic inequalities in health states were consistently found across SEP indicators in both genders, but those for occupational groups were minimal or reversed. Socioeconomic inequalities in most NCDs, such as diabetes, hypertension, IHD and arthritis, apart from cancer, were significant in women, but not in men. Few socioeconomic inequalities were observed in HBV and injury.

**Figure 1:**
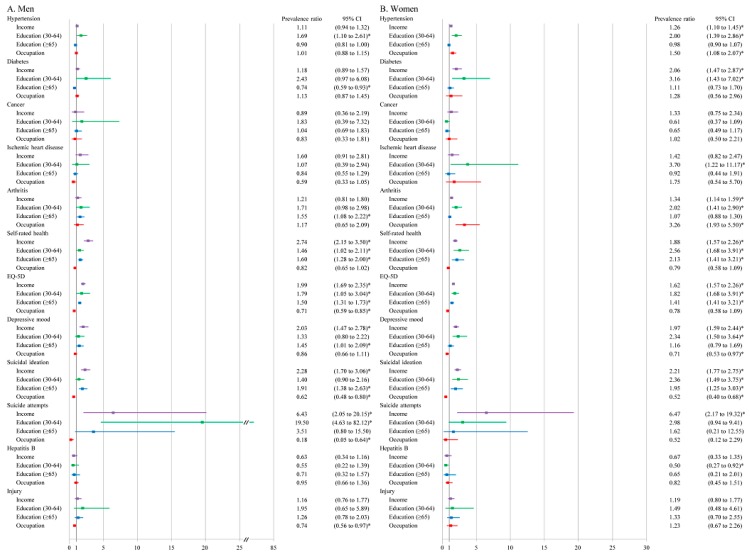
Age-adjusted prevalence ratios of 12 health problems in men (**A**) and women (**B**) according to income, education, and occupational groups in KNHANES 2010–2012. Notes: KNHANES = Korea National Health and Nutrition Examination Survey, EQ-5D = EuroQol five-dimension questionnaire. * denotes statistical significance at the 95% confidence level. †Reference categories of SEP measures are highest income quartile for income, college graduated and above for education of the middle aged (30–64), high school graduate and above for education of elderly people (≥65), and manager and office workers for occupational class.

Among SEP measures, associations of income and education with health problems were in a similar direction, while those of the occupational group were often reversed, particularly for mental and subjective health. For example, the manual worker to manager/office worker PRs for self-rated health and suicide ideation were 0.82 (95% CI: 0.65–1.02) and 0.62 (0.48–0.80) in men and 0.79 (0.80–0.96) and 0.52 (0.40–0.68), in women, respectively. For some NCDs (diabetes, hypertension, cancer, and IHD), reversed inequalities were also observed with respect to educational attainment among the elderly (Figure 1).

## 4. Discussion

Socioeconomic inequalities across 12 health problems showed similar patterns among similar entities of health conditions such as NCDs, mental health and subjective health states. In general, the largest socioeconomic inequalities were observed for mental health (i.e., depression, suicidal ideation, and suicide attempts), and significant socioeconomic associations were also observed for subjective health states (i.e., self-rated health and quality of life). Most NCDs (i.e., diabetes, hypertension, IHD, and arthritis) commonly showed larger socioeconomic inequalities among women than men. No, or only small, inequalities were observed for cancer, injuries, and HBV infection. Health inequalities were similarly observed across SEP measures but, for occupation, the association was often reversed.

### 4.1. Comparison with Previous Studies

#### 4.1.1. Socioeconomic Inequalities in NCDs and Mental Health and Gender Differences

Socioeconomic associations for the NCDs, including diabetes, hypertension, IHD, and arthritis, showed similar directions and magnitudes and were apparent mostly among women. This finding is in line with some studies conducted in Asian and developing countries [12,14], including Korea [13,25], which reported no or inconsistent patterns of socioeconomic inequalities for these diseases, although other studies, mostly from advanced Western countries [6,10,11], reported clear gradients with respect to socioeconomic position. An explanation for the greater socioeconomic inequality for NCDs among women, as described previously [5], is that, in general, some common risk factors for NCDs, such as physical activity, obesity, and work-related exposure (e.g., job stress and long working hours) [26,27] exhibit more obvious socioeconomic patterns among women than men in Korea. In addition, it is known that those from upper SEPs are more likely to attend CVD screening, and more chances of early detection, particularly among men [28,29], which leads to masking socioeconomic inequalities. Selective survival (i.e., those of higher SEP exhibit lower mortality rates because of prolonged survival) could be the primary reason for our finding of reversed educational inequalities in certain NCDs among the elderly. That is, the strong socioeconomic differences in mortality among the deceased may be an explanation for the weak or even reversed socioeconomic differences in morbidity among elderly survivors [30,31], though the current study cannot establish this relationship because of the cross-sectional nature of the data.

One key finding of this study is the large and consistent socioeconomic inequalities in mental illness observed for depression, suicidal ideation and suicide attempts. Previous longitudinal studies have also reported that the strong accumulation of socioeconomic adversity among patients with these mental illnesses [32,33] often precede suicide. This suggests that socioeconomically-driven motives substantially form a basis of suicide, for which Korea has the highest rate among OECD countries [34].

#### 4.1.2. Socioeconomic Inequalities in Cancer, Injuries, and Communicable Diseases

In general, the influences of socioeconomic factors on cancer, injuries and HBV infection were minimal in this study. The general lack of socioeconomic inequalities in terms of cancer was consistent with previous studies [5,35]. This may be due to ignorance of cancer subtypes. Pooling all cancer subtypes into one category clearly results in summing of inconsistent patterns in cancer inequalities, e.g., marked gradient for trachea, bronchus, and lung cancers, but no significant gradient for breast cancer [35]. Similarly, if socioeconomic inequalities in injuries are assessed by injury subcategory, substantial gradients in the magnitude of the association may be apparent; e.g., emerging evidence shows greater health inequalities for fatal injuries [15] and childhood injuries [36]. Evidence suggests that not all communicable diseases are related to socioeconomic inequalities in European countries, although inequalities in emerging infections and infections endemic to certain populations were evident [2]. The weak socioeconomic association in communicable diseases observed here and in other studies may be mainly due to an increase in the coverage of certain vaccinations [37]. Korea adopted a universal HBV vaccination program in 1983 for all neonates, which resulted in a decreased sero-prevalence (3.7% in 2007) and decreased socioeconomic inequalities [38]. In Korea, the major type of national health insurance payment is fee-for-service plan and, accordingly, insurance coverage is heterogeneous for prevention, screening, diagnosis, and outpatient and inpatient care across diseases. For example, the critical illness insurance scheme launched by Korean government was designed to relieve the financial strain of patients with one of the four major target diseases (i.e., cancer, severe burn, cerebrovascular, cardiac and rare and incurable diseases) by providing special benefits. Further, despite the universal health care system, Korea records the highest level of out-of-pocket expenditure [34]. Thus, the small or non-existent gradients for some health conditions, such as HBV infection, may indicate that fairer access to health care can be a way to alleviate socioeconomic burden.

#### 4.1.3. Socioeconomic Inequalities in Health States and Differences in Three SEP Measures

Socioeconomic inequalities were also evident in health states (e.g., self-reported health and quality of life) [4]. This suggests that Korea’s highest proportion of poor self-reported health problems among OECD countries [34] may need to account for the strongly socially patterned poor health prevalence. Reversal of inequalities by occupation-based measure dissimilar to associations of income and education were observed among indicators of subjective health states and mental health, which also supports closeness of health inequality patterns among similar entities of health conditions. This may indicate that manual vs. non-manual dichotomy become less relevant for inequalities in some health conditions with changes in labour market structure; increase in atypical job in service industry and the decrease in unskilled and semi-skilled manual jobs [39]. Increase in mental workload, particularly among higher occupational position, may contribute to the reversed relationship for mental and subjective health states [40].

#### 4.1.4. Implications of Similarities and Differences of Health Inequalities among 12 Health Conditions

Overall, the inequalities observed among the 12 health problems varied and the health inequality patterns were relatively similar among a group of health problems (e.g., NCDs, mental health problems and subjective health states). Given the fact that the health inequalities in our study are measured according to prevalence, the health inequalities may be the net result of many different converging and diverging factors along the course of illness (i.e., disease prevention, diagnosis, care quality, chance of survival and consequences of illness), which constitute a unique context of each health problem. This implies that health inequalities varied partly because of behavioural risk factors and health care use, which were linked to specific health problems. In line with this, obvious socioeconomic gradients in health-related behaviours [26] and health care use [28] among women may constitute sources for socioeconomic differences in NCD prevalence between men and women. Future studies are required to ensure a firm foundation on how the extents of socioeconomic differences along the stages of disease courses contribute to the stronger socioeconomic inequalities in women than men observed for NCDs. However, when addressing health inequalities in the connection with health–related behaviours and health care use, caution is required to not be limited to a narrowly-focused individualistic approach (e.g., media campaigns for smoking cessation [41]). Universal approaches, such as mass catering in school [42], mandatory seat belt use [43], or reducing out-of-pocket spending for equalized accessibility to quality care [44], in combination with targeted approaches toward deprived populations or areas may facilitate more complete strategies to reduce health inequalities [45,46].

### 4.2. Methodological Consideration

A major strength of this study was the comprehensive inclusion of diseases for health inequality comparisons. Based on the variation in the spectrum of health domains, we found that the extent of health inequalities differed among the domains. This also suggests a direction for future studies and it will be necessary to include other dimensions of health conditions, such as mild versus severe, acute versus chronic, and fatal versus non-fatal conditions to further clarify the origins of health inequalities in relation to characteristics of health condition. Second, the use of a single nationally-representative dataset enabled an approach for assessing multiple health problems without involving the common limitations of comparative studies, such as heterogeneous samples and non-standardized socioeconomic measures.

This study had several limitations. First, it should be noted that the measure used here was prevalence, which does not distinguish between incidence and survival. Prevalence is of value for summarizing the current health status and has an independent message. Health inequalities measured according to prevalence may be more reflective of incidence in some diseases (e.g., injuries and infections) and more reflective of survival in other long-term diseases (e.g., hypertension and diabetes). Further longitudinal studies to elaborate on the differences in health inequalities must distinguish among incidence, survival, and prevalence. Second, some of the data were self-reported and were, therefore, subject to misclassification. Disease prevalence diagnosed with the aid of laboratory data, such as those of hypertension, diabetes, and hepatitis B, are relatively accurate. In comparison, the prevalence of arthritis, cancer, and IHD, which are measured only by self-reporting of a physician’s diagnosis, are likely to be less accurate. In particular, the use of self-reported measures for depression can lead to biases due to either recall or perceived stigma. Considering the lower accessibility to mental health services and under-reporting of mental illness among those with lower SEP, the results of this study obtained from self-reported data may have caused underestimation of socioeconomic inequalities in these conditions. Third, the sample size of the suicide attempt group was small and, therefore, the age-adjusted prevalence and PRs had relatively wide confidence intervals. However, it is meaningful that suicide attempts also showed apparent gaps among SEPs, similar to the other mental health problems. Fourth, in the current study, to describe the magnitude of health inequalities, age-adjusted prevalence ratios (PRs) and prevalence differences (PDs) were calculated by comparing the lowest and highest SEP groups. Though this method enables demonstrating social group differences, it cannot capture total health inequalities among individuals [47]. Thus, to measure socioeconomic variation in health within a population, future studies on health inequalities needed to be complemented by recent advances of total inequality approaches, such as rank-dependent and level-dependent health inequalities indicators [48].

## 5. Conclusions

Health inequalities varied across 12 health problems and the patterns of inequalities were similar among a group of health problems. This finding suggests that the patterns in health inequalities were attributable to socioeconomic and health care-related characteristics along the course of occurrence and management of health problems. Monitoring of the magnitudes of socioeconomic inequalities across health problems could help to identify which conditions to target. In the current study, mental illnesses appeared to require prioritization of socioeconomic approaches for improvement in terms of absolute prevalence and relative socioeconomic distribution.

## Figures and Tables

**Table 1:** Sociodemographic characteristics and health conditions and states of the study participants in KNHANES 2010–2012.

	Men	Women		Total
	N (%)	N (%)	*p*-Value ^†^	N (%)
Number of individuals	7515 (43.5)	9777 (56.5)		17,292 (100)
Age				
30–64	5484 (73.0)	7066 (72.3)		12,550 (72.6)
≥65	2031 (27.0)	2711 (27.7)	0.3048	4742 (27.4)
Income				
Low	1416 (19.1)	2221 (23.1)		3637 (21.4)
Mid-low	1934 (26.1)	2504 (26.0)		4438 (26.1)
Mid-high	2013 (27.2)	2460 (25.6)		4473 (26.3)
High	2041 (27.6)	2435 (25.3)	<0.001	4476 (26.3)
Education				
Age (30–64)				
Low	500 (10.3)	1269 (19.2)		1769 (15.4)
Middle	2281 (46.8)	3296 (49.7)		5577 (48.5)
High	2095 (43.0)	2062 (31.1)	<0.001	4157 (36.1)
Age (≥65)				
Low	837 (45.5)	1996 (81.9)		2833 (66.2)
Middle	316 (17.2)	206 (8.5)		522 (12.2)
High	688 (37.4)	234 (9.6)	<0.001	922 (21.6)
Occupation				
Manager or office job	1827 (35.6)	1162 (28.0)		2989 (32.2)
Service or sales	687 (13.4)	1183 (28.5)		1870 (20.2)
Manual work	2618 (51.0)	1801 (43.4)	<0.001	4419 (47.6)
Diabetes				
Yes	878 (14.0)	822 (9.8)		1700 (11.6)
No	5394 (86.0)	7561 (90.2)	<0.001	12,955 (88.4)
Hypertension				
Yes	2493 (37.3)	2956 (32.7)		5449 (34.6)
No	4192 (62.7)	6097 (67.4)	<0.001	10,289 (65.4)
Cancer				
Yes	74 (1.1)	157 (1.7)		231 (1.5)
No	6648 (98.9)	8926 (98.3)	<0.001	15,574 (98.5)
Ischemic heart disease				
Yes	251 (3.7)	252 (2.8)		503 (3.2)
No	6472 (96.3)	8834 (97.2)	0.3501	15,306 (96.8)
Arthritis				
Yes	361 (9.1)	1813 (33.9)		2714 (23.3)
No	3629 (91.0)	3528 (66.1)	<0.001	7157 (76.7)
Self-rated health status				
Good	5595 (83.2)	6951 (76.5)		12,546 (79.3)
Poor	1129 (16.8)	2137 (23.5)	<0.001	3266 (20.7)
EQ-5D				
Below median	2613 (34.8)	4497 (46.0)		7110 (41.1)
Above median	4902 (65.2)	5280 (54.0)	<0.001	10,182 (58.9)
Depressive mood				
Yes	627 (9.3)	1517 (16.7)		2144 (13.6)
No	6098 (90.7)	7543 (83.3)	<0.001	13,641 (86.4)
Suicide ideation				
Yes	711 (10.6)	1612 (17.8)		2323 (14.7)
No	6017 (89.4)	7447 (82.2)	<0.001	13,460 (85.3)
Suicide attempts				
Yes	51 (0.8)	65 (0.7)		116 (0.7)
No	6673 (99.2)	8991 (99.3)	0.7671	15,664 (99.3)
Hepatitis B				
Yes	287 (4.3)	303 (3.5)		590 (3.8)
No	6379 (95.7)	8436 (96.5)	0.0072	14,815 (96.2)
Injury experience				
Yes	468 (7.0)	601 (6.6)		1069 (6.8)
No	6250 (93.0)	8472 (93.4)	0.3970	14,722 (93.2)

^†^ The *p*-values were calculated from the chi-square test of differences between men and women. Notes: KNHANES = Korea National Health and Nutrition Examination Survey, EQ-5D = EuroQol five-dimension questionnaire. %: row percent apart from number of individuals. *p*-value was obtained from the chi-square test.

## Supplementary Materials

The following are available online at http://www.mdpi.com/1660-4601/15/12/2868/s1, **Table S1.** Age-adjusted prevalence and prevalence ratios of 12 health conditions in total participants according to education, income, and occupational groups in KNHANES 2010–2012, **Table S2.** Age-adjusted prevalence and prevalence ratios of 12 health conditions in men according to education, income, and occupational groups in KNHANES 2010–2012, **Table S3.** Age-adjusted prevalence and prevalence ratios of 12 health conditions in women according to education, income, and occupational groups in KNHANES 2010–2012, **Table S4.** Age-adjusted prevalence and prevalence differences of 12 health conditions in total participants according to education, income, and occupational groups in KNHANES 2010-2012, **Table S5.** Age-adjusted prevalence and prevalence differences of 12 health conditions in men according to education, income, and occupational groups in KNHANES 2010–2012, **Table S6.** Age-adjusted prevalence and prevalence differences of 12 health conditions in women according to education, income, and occupational groups in KNHANES 2010–2012.
