# Supplementary material for: An Assessment of Magnitudes and Patterns of Socioeconomic Inequalities across Various Health Problems: A Large National Cross-Sectional Survey in Korea
Source: Int J Environ Res Public Health. 2018 Dec 14;15(12):2868. doi: 10.3390/ijerph15122868 (PMC6313447; doi:10.3390/ijerph15122868)
Supplement: Supplementary file 1 [file ijerph-15-02868-s001.pdf]

Table S1. Age-adjusted prevalences and prevalence ratios of 12 health conditions in total participants according to education, income and occupational groups in KNHANES 2010-2012.

		Hypertension				Diabetes				Cancer				Ischemic heart disease			
		N	Age standardized prevalence	PR *	95% CI	N	Age standardized prevalence	PR *	95% CI	N	Age standardized prevalence	PR *	95% CI	N	Age standardized prevalence	PR *	95% CI
Income	Low	3,301	30.35	1.16	(1.04, 1.30)	2,867	12.65	1.43	(1.15, 1.78)	3,307	2.87	1.23	(0.76, 1.98)	3,309	2.69	1.39	(0.92, 2.12)
	Mid-low	4,033	27.72	1.06	(0.97, 1.16)	3,767	9.18	1.04	(0.88, 1.22)	4,050	2.24	0.96	(0.68, 1.36)	4,051	1.85	0.96	(0.66, 1.40)
	Mid-high	4,107	27.81	1.06	(0.98, 1.16)	3,908	9.19	1.04	(1.19, 1.24)	4,126	2.17	0.93	(0.66, 1.30)	4,127	2.11	1.09	(0.74, 1.62)
	High	4,123	26.15	ref		3,961	8.86	ref		4,148	2.34	ref		4,148	1.93	ref	
Education (30-64)	Low	1,761	29.04	1.55	(1.14, 2.10)	1,664	12.46	2.21	(1.18, 4.16)	1,768	1.67	1.16	(0.53, 2.48)	1,769	1.4	1.54	(0.78, 3.04)
	Middle	5,545	20.69	1.1	(0.99, 1.23)	5,297	7.46	1.32	(1.06, 1.65)	5,577	1.77	1.24	(0.88, 1.87)	5,577	0.97	1.06	(0.61, 1.83)
	High	4,133	18.75	ref		3,969	5.63	ref		4,156	1.43	ref		4,157	0.91	ref	
Education (≥65)	Low	2,826	63.87	1.08	(0.99, 1.17)	2,411	20.63	0.97	(0.79, 1.18)	2,833	4.5	0.76	(0.57, 1.20)	2,832	6.28	0.84	(0.57, 1.25)
	Middle	522	58.56	0.99	(0.89, 1.10)	458	24.54	1.15	(0.89, 1.49)	522	6.08	1.03	(0.65, 1.80)	522	8.52	1.15	(0.74, 1.76)
	High	920	59.28	ref		835	21.34	ref		922	5.9	ref		922	7.44	ref	
Occupation (30-64)	Physical work	3,156	23.07	1.09	(0.97, 1.23)	3,029	7.63	1.12	(0.88, 1.44)	3,175	0.94	0.9	(0.52, 1.54)	3,176	1.01	0.68	(0.40, 1.15)
	Service or sales	1,723	21.08	1	(0.86, 1.15)	1,675	6.95	1.02	(0.77, 1.36)	1,735	1.33	1.28	(0.71, 2.24)	1,735	0.86	0.58	(0.28, 1.18)
	Manager or office job	2,877	21.12	ref		2,766	6.79	ref		2,895	1.04	ref		2,895	1.49	ref	

Notes: KNHANES = Korea National Health and Nutrition Examination Survey, EQ-5D = EuroQol five-dimension questionnaire, PR = Prevalence Ratios.

Table S 1. Age-adjusted prevalences and prevalence ratios of 12 health conditions in total participants according to education, income and occupational groups in KNHANES 2010-2012. (continued)

		Arthritis				Self-rated health				EQ-5D				Depressive mood			
		N	Age standardized prevalence	PR *	95% CI	N	Age standardized prevalence	PR *	95% CI	N	Age standardized prevalence	PR *	95% CI	N	Age standardized prevalence	PR *	95% CI
Income	Low	2,913	20.75	1.45	(1.23, 1.70)	3,312	30.01	2.21	(1.89, 2.58)	3,479	48.22	1.77	(1.60, 1.95)	3,291	22.39	2.04	(1.69, 2.45)
	Mid-low	2,376	16.91	1.18	(1.01, 1.38)	4,050	18.15	1.33	(1.18, 1.51)	4,204	33.25	1.22	(1.13, 1.32)	4,046	13.47	1.23	(1.05, 1.43)
	Mid-high	1,894	16.06	1.12	(0.94, 1.34)	4,128	14.84	1.09	(0.96, 1.25)	4,248	29.75	1.09	(1.01, 1.18)	4,130	10.57	0.96	(0.82, 1.13)
	High	2,032	14.33	ref		4,148	13.6	ref		4,264	27.26	ref		4,147	10.98	ref	
Education (30-64)	Low	1,634	17.25	2.83	(2.04, 3.92)	1,769	26.24	2.34	(1.62, 3.38)	1,769	41.63	2.06	(1.60, 2.67)	1,764	24.25	2.53	(1.64, 3.91)
	Middle	2,614	10.03	1.64	(1.17, 2.30)	5,577	14.44	1.29	(1.11, 1.49)	5,577	25.43	1.26	(1.15, 1.39)	5,572	13.12	1.37	(1.18, 1.59)
	High	778	6.1	ref		4,157	11.21	ref		4,157	20.16	ref		4,153	9.58	ref	
Education (≥65)	Low	2,832	35.58	2.34	(1.88, 2.91)	2,832	39.01	2	(1.66, 2.41)	2,833	64.2	1.65	(1.48, 1.85)	2,818	17.75	1.68	(1.32, 2.16)
	Middle	522	27.04	1.78	(1.36, 2.33)	522	30.58	1.57	(1.23, 2.00)	522	55.14	1.42	(1.23, 1.64)	519	12.7	1.2	(0.85, 1.71)
	High	922	15.21	ref		922	19.52	ref		922	38.81	ref		920	10.54	ref	
Occupation (30-64)	Physical work	1,842	9.88	2.14	(1.41, 3.25)	2,989	11.67	0.75	(0.62, 0.90)	2,989	19.79	0.72	(0.63, 0.82)	2,987	9.11	0.79	(0.65, 0.96)
	Service or sales	784	12.45	2.7	(1.74, 4.18)	1,870	16.62	1.07	(0.90, 1.26)	1,870	27.57	1	(0.90, 1.11)	1,867	12.64	1.09	(0.90, 1.32)
	Manager or office job	653	4.61	ref		4,418	15.59	ref		4,419	27.58	ref		4,415	11.56	ref	

Notes: KNHANES = Korea National Health and Nutrition Examination Survey, EQ-5D = EuroQol five-dimension questionnaire, PR = Prevalence Ratios.

Table S1. Age-adjusted prevalences and prevalence ratios of 12 health conditions in total participants according to education, income and occupational groups in KNHANES 2010-2012. (continued)

Total		Suicidal ideation				Suicide attempts				Hepatitis B				Injury			
		N	Age standardized prevalence	PR *	95% CI	N	Age standardized prevalence	PR *	95% CI	N	Age standardized prevalence	PR *	95% CI	N	Age standardized prevalence	PR *	95% CI
Income	Low	3,290	23.65	2.3	(1.92, 2.75)	3,289	2.34	6.5	(2.98, 14.21)	3,077	3.11	0.65	(0.41, 1.03)	3,300	7.57	1.15	(0.86, 1.54)
	Mid-low	4,046	14.38	1.4	(1.20, 1.63)	4,046	0.67	1.87	(0.83, 4.18)	3,955	3.97	0.83	(0.63, 1.08)	4,046	6.72	1.02	(0.81, 1.28)
	Mid-high	4,130	11.77	1.14	(0.97, 1.35)	4,128	0.44	1.23	(0.51, 2.98)	4,069	4.24	0.88	(0.68, 1.15)	4,126	7.15	1.08	(0.88, 1.34)
	High	4,146	10.28	ref		4,146	0.36	ref		4,104	4.8	ref		4,147	6.59	ref	
Education (30-64)	Low	1,764	22.15	2.36	(1.53, 3.65)	1,763	2.05	8.92	(3.10, 25.61)	1,699	2.13	0.52	(0.31, 0.88)	1,769	10.23	1.59	(0.72, 3.52)
	Middle	5,572	12.29	1.31	(1.13, 1.52)	5,571	0.76	3.32	(1.42, 7.76)	5,414	4.09	1	(0.77, 1.30)	5,577	7.11	1.11	(0.90, 1.36)
	High	4,152	9.39	ref		4,152	0.23	ref		4,034	4.08	ref		4,157	6.43	ref	
Education (≥65)	Low	2,818	26.36	2.34	(1.84, 2.98)	2,818	0.97	2.37	(0.67, 8.31)	2,484	1.54	0.78	(0.41, 1.50)	2,833	8.44	1.28	(0.90, 1.82)
	Middle	518	14.96	1.33	(0.94, 1.88)	518	1.48	3.6	(0.90, 14.33)	471	1.34	0.68	(0.28, 1.61)	522	6.26	0.95	(0.58, 1.56)
	High	920	11.26	ref		920	0.41	ref		852	1.97	ref		922	6.6	ref	
Occupation (30-64)	Physical work	2,987	7.68	0.6	(0.50, 0.73)	2,987	0.17	0.27	(0.10, 0.72)	2,896	4.49	0.94	(0.68, 1.29)	2,989	6.25	0.8	(0.62, 1.03)
	Service or sales	1,866	13.71	1.08	(0.90, 1.29)	1,865	1.03	1.66	(0.63, 4.35)	1,820	4.4	0.92	(0.64, 1.31)	1,870	6.84	0.87	(0.67, 1.13)
	Manager or office job	4,414	12.72	ref		4,414	0.62	ref		4,274	4.8	ref		4,419	7.85	ref	

Notes: KNHANES = Korea National Health and Nutrition Examination Survey, EQ-5D = EuroQol five-dimension questionnaire, PR = Prevalence Ratios.

Table S2. Age-adjusted prevalences and prevalence ratios of 12 health conditions in men according to education, income and occupational groups in KNHANES 2010-2012.

		Hypertension				Diabetes				Cancer				Ischemic heart disease			
		N	Age standardized prevalence	PR *	95% CI	N	Age standardized prevalence	PR *	95% CI	N	Age standardized prevalence	PR *	95% CI	N	Age standardized prevalence	PR *	95% CI
Income	Low	1,259	33.2	1.11	(0.94, 1.32)	1,101	14.1	1.18	(0.89, 1.57)	1,260	1.6	0.89	(0.36, 2.19)	1,261	3.23	1.6	(0.91, 2.81)
	Mid-low	1,714	31.12	1.04	(0.92, 1.17)	1,608	9.88	0.83	(0.66, 1.04)	1,724	1.54	0.86	(0.44, 1.68)	1,724	1.86	0.92	(0.57, 1.48)
	Mid-high	1,811	30.71	1.03	(0.91, 1.15)	1,726	10.55	0.88	(0.70, 1.12)	1,823	1.5	0.84	(0.44, 1.59)	1,823	2.48	1.23	(0.76, 1.98)
	High	1,832	29.93	ref		1,772	11.93	ref		1,823	1.79	ref		1,846	2.03	ref	
Education (30-64)	Low	497	41.92	1.69	(1.10, 2.61)	469	17.9	2.43	(0.97, 6.08)	500	1.81	1.83	(0.39, 7.32)	500	1.55	1.07	(0.39, 2.94)
	Middle	2,266	28.13	1.14	(1.01, 1.28)	2,175	9.65	1.31	(1.04, 1.65)	2,281	0.71	0.72	(0.35, 1.43)	2,281	1.5	1.04	(0.62, 1.75)
	High	2,078	24.76	ref		1,997	7.37	ref		2,095	0.99	ref		2,095	1.45	ref	
Education (≥65)	Low	835	53.52	0.9	(0.81, 1.00)	723	18.39	0.74	(0.59, 0.93)	837	5.31	1.04	(0.69, 1.83)	837	6.79	0.84	(0.55, 1.29)
	Middle	316	57.85	0.98	(0.86, 1.11)	281	28.55	1.15	(0.85, 1.55)	316	4.11	0.8	(0.40, 1.76)	316	9.38	1.16	(0.71, 1.89)
	High	686	59.29	ref		624	24.91	ref		688	5.13	ref		688	8.1	ref	
Occupation (30-64)	Physical work	1,930	25.33	1.01	(0.88, 1.15)	1,854	8.77	1.13	(0.87, 1.45)	1,943	0.62	0.83	(0.33, 1.81)	1,943	1.03	0.59	(0.33, 1.05)
	Service or sales	631	25.88	1.03	(0.87, 1.22)	621	9.94	1.28	(0.93, 1.75)	638	0.32	0.43	(0.09, 1.54)	638	0.9	0.52	(0.22, 1.22)
	Manager or office job	1,736	25.2	ref		1,665	7.78	ref		1,750	0.75	ref		1,750	1.74	ref	

Notes: KNHANES = Korea National Health and Nutrition Examination Survey, EQ-5D = EuroQol five-dimension questionnaire, PR = Prevalence Ratios.

Table S2. Age-adjusted prevalences and prevalence ratios of 12 health conditions in men according to education, income and occupational groups in KNHANES 2010-2012. (continued)

		Arthritis				Self-rated health				EQ-5D				Depressive mood			
		N	Age standardized prevalence	PR *	95% CI	N	Age standardized prevalence	PR *	95% CI	N	Age standardized prevalence	PR *	95% CI	N	Age standardized prevalence	PR *	95% CI
Income	Low	1,097	7.15	1.21	(0.81, 1.80)	1,262	28.48	2.74	(2.15, 3.50)	1,339	41.99	1.99	(1.69, 2.35)	1,255	16.84	2.03	(1.47, 2.78)
	Mid-low	1,033	7.22	1.22	(0.84, 1.76)	1,724	15.71	1.51	(1.24, 1.85)	1,799	28.46	1.35	(1.17, 1.55)	1,725	9.38	1.13	(0.86, 1.48)
	Mid-high	862	5.42	0.91	(0.60, 1.40)	1,823	12.13	1.17	(0.94, 1.45)	1,886	23.34	1.11	(0.95, 1.28)	1,826	7.36	0.88	(0.67, 1.17)
	High	952	5.92	ref		1,846	10.38	ref		1,912	21.08	ref		1,849	8.31	ref	
Education (30-64)	Low	457	2.52	1.71	(0.98, 2.98)	500	15.69	1.46	(1.02, 2.11)	500	29.18	1.79	(1.05, 3.04)	500	9.71	1.33	(0.80, 2.22)
	Middle	1,189	1.98	1.35	(0.81, 2.24)	2,281	12.48	1.16	(0.95, 1.43)	2,281	20.89	1.28	(1.09, 1.50)	2,279	10.27	1.41	(1.10, 1.81)
	High	497	1.47	ref		2,095	10.72	ref		2,095	16.32	ref		2,094	7.28	ref	
Education (≥65)	Low	837	13.16	1.55	(1.08, 2.22)	837	31.33	1.6	(1.28, 2.00)	837	53.92	1.5	(1.31, 1.73)	832	12.34	1.45	(1.01, 2.09)
	Middle	316	15.36	1.81	(1.19, 2.76)	316	24.64	1.26	(0.93, 1.71)	316	45.8	1.28	(1.06, 1.54)	314	10.61	1.25	(0.79, 1.97)
	High	688	8.48	ref		688	19.57	ref		688	35.86	ref		686	8.49	ref	
Occupation (30-64)	Physical work	1,051	4.81	1.17	(0.65, 2.09)	1,827	10.55	0.82	(0.65, 1.02)	1,827	16.92	0.71	(0.59, 0.85)	1,826	7.74	0.86	(0.66, 1.11)
	Service or sales	240	2.71	0.66	(0.27, 1.60)	687	13.25	1.02	(0.77, 1.37)	687	19.01	0.8	(0.64, 0.99)	687	8.76	0.97	(0.70, 1.34)
	Manager or office job	477	4.13	ref		2,618	12.93	ref	(2.15, 3.50)	2,618	23.84	ref		2,616	9.04	ref	

Notes: KNHANES = Korea National Health and Nutrition Examination Survey, EQ-5D = EuroQol five-dimension questionnaire, PR = Prevalence Ratios.

Table S2. Age-adjusted prevalences and prevalence ratios of 12 health conditions in men according to education, income and occupational groups in KNHANES 2010-2012. (continued)

		Suicidal ideation				Suicide attempts				Hepatitis B				Injury			
		N	Age standardized prevalence	PR *	95% CI	N	Age standardized prevalence	PR *	95% CI	N	Age standardized prevalence	PR *	95% CI	N	Age standardized prevalence	PR *	95% CI
Income	Low	1,255	18.54	2.28	(1.70, 3.06)	1,255	2.32	6.43	(2.05, 20.15)	1,203	3.26	0.63	(0.34, 1.16)	1,257	7.46	1.16	(0.76, 1.77)
	Mid-low	1,725	11	1.36	(1.05, 1.75)	1,725	0.6	1.66	(0.53, 5.25)	1,704	4.21	0.81	(0.56, 1.16)	1,723	8.28	1.29	(0.94, 1.75)
	Mid-high	1,826	8.04	0.99	(0.75, 1.31)	1,826	0.17	0.47	(0.06, 3.60)	1,819	4.76	0.91	(0.64, 1.29)	1,823	8.04	1.25	(0.93, 1.68)
	High	1,848	8.12	ref		1,848	0.36	ref		1,853	5.22	ref		1,846	6.44	ref	
Education (30-64)	Low	500	10.51	1.4	(0.90, 2.16)	500	3.85	19.5	(4.63, 82.12)	481	2.67	0.55	(0.22, 1.39)	500	13.42	1.95	(0.65, 5.89)
	Middle	2,279	10.01	1.33	(1.04, 1.70)	2,279	0.51	2.58	(0.63, 10.67)	2,235	5.27	1.08	(0.78, 1.50)	2,281	9	1.31	(1.00, 1.71)
	High	2,094	7.52	ref		2,094	0.2	ref		2,041	4.87	ref		2,095	6.87	ref	
Education (≥65)	Low	832	19.51	1.91	(1.38, 2.63)	832	1.5	3.51	(0.80, 15.50)	751	1.64	0.71	(0.32, 1.57)	837	8.4	1.26	(0.78, 2.03)
	Middle	313	16.19	1.58	(1.02, 2.45)	313	1.66	3.9	(0.82, 18.60)	289	2.29	0.99	(0.39, 2.52)	316	4.98	0.75	(0.38, 1.45)
	High	686	10.23	ref		686	0.43	ref		639	2.31	ref		688	6.69	ref	
Occupation (30-64)	Physical work	1,826	6.39	0.62	(0.48, 0.80)	1,826	0.12	0.18	(0.05, 0.64)	1,770	4.95	0.95	(0.66, 1.36)	1,827	6.24	0.74	(0.56, 0.97)
	Service or sales	687	9.43	0.91	(0.67, 1.24)	687	0.56	0.85	(0.21, 3.48)	673	4.89	0.94	(0.58, 1.51)	687	8.1	0.95	(0.65, 1.39)
	Manager or office job	2,615	10.33	ref		2,615	0.66	ref		2,549	5.22	ref		2,618	8.49	ref	

Notes: KNHANES = Korea National Health and Nutrition Examination Survey, EQ-5D = EuroQol five-dimension questionnaire, PR = Prevalence Ratios.

Table S3. Age-adjusted prevalences and prevalence ratios of 12 health conditions in women according to education, income and occupational groups in KNHANES 2010-2012.

		Hypertension				Diabetes				Cancer				Ischemic heart disease			
		N	Age standardized prevalence	PR *	95% CI	N	Age standardized prevalence	PR *	95% CI	N	Age standardized prevalence	PR *	95% CI	N	Age standardized prevalence	PR *	95% CI
Income	Low	2,042	27.11	1.26	(1.10, 1.45)	1,766	11.55	2.06	(1.47, 2.87)	2,047	4.15	1.33	(0.75, 2.34)	2,048	2.27	1.42	(0.82, 2.47)
	Mid-low	2,319	24.52	1.14	(1.02, 1.28)	2,159	8.64	1.54	(1.18, 2.01)	2,326	2.83	0.91	(0.62, 1.33)	2,327	1.85	1.16	(0.67, 2.03)
	Mid-high	2,296	24.28	1.13	(1.01, 1.27)	2,182	7.74	1.38	(1.03, 1.85)	2,303	2.97	0.95	(0.65, 1.39)	2,304	1.75	1.1	(0.60, 2.01)
	High	2,291	21.45	ref		2,189	5.61	ref		2,302	3.13	ref		2,302	1.6	ref	
Education (30-64)	Low	730	38.55	2	(1.39, 2.86)	1,195	10.69	3.16	(1.43, 7.02)	1,268	1.41	0.61	(0.37, 1.09)	1,269	1.49	3.7	(1.22, 11.17)
	Middle	1,457	28.02	1.45	(1.12, 1.87)	3,122	5.52	1.64	(1.05, 2.55)	3,296	3.01	1.29	(0.95, 2.09)	3,296	0.74	1.84	(0.59, 5.77)
	High	749	19.31	ref		1,972	3.38	ref		2,061	2.33	ref		2,062	0.4	ref	
Education (≥65)	Low	1,445	85.13	0.98	(0.90, 1.07)	1,688	21.75	1.11	(0.73, 1.70)	1,996	4.59	0.65	(0.49, 1.17)	1,995	6.35	0.92	(0.44, 1.91)
	Middle	144	88.35	1.02	(0.92, 1.13)	177	18.88	0.96	(0.55, 1.68)	206	10.74	1.52	(0.83, 3.31)	206	9.34	1.35	(0.56, 3.26)
	High	155	86.59	ref		211	19.59	ref		234	7.08	ref		234	6.89	ref	
Occupation (30-64)	Physical work	1,226	17.27	1.5	(1.08, 2.07)	1,175	5.29	1.28	(0.56, 2.96)	1,232	1.74	1.02	(0.50, 2.21)	1,233	0.93	1.75	(0.54, 5.70)
	Service or sales	1,092	16.85	1.46	(1.05, 2.03)	1,054	4.76	1.16	(0.49, 2.71)	1,097	2.05	1.2	(0.66, 2.42)	1,097	0.81	1.53	(0.39, 5.94)
	Manager or office job	1,141	11.53	ref		1,101	4.12	ref		1,145	1.71	ref		1,145	0.53	ref	

Notes: KNHANES = Korea National Health and Nutrition Examination Survey, EQ-5D = EuroQol five-dimension questionnaire, PR = Prevalence Ratios.

Table S3. Age-adjusted prevalences and prevalence ratios of 12 health conditions in women according to education, income and occupational groups in KNHANES 2010-2012. (continued)

		Arthritis				Self-rated health				EQ-5D				Depressive mood			
		N	Age standardized prevalence	PR *	95% CI	N	Age standardized prevalence	PR *	95% CI	N	Age standardized prevalence	PR *	95% CI	N	Age standardized prevalence	PR *	95% CI
Income	Low	1,816	30.13	1.34	(1.14, 1.59)	2,050	30.76	1.88	(1.57, 2.26)	2,140	53.41	1.62	(1.57, 2.26)	2,036	26.96	1.97	(1.59, 2.44)
	Mid-low	1,343	25.18	1.12	(0.95, 1.33)	2,326	20.29	1.24	(1.07, 1.44)	2,405	37.65	1.14	(1.07, 1.44)	2,321	17.2	1.26	(1.05, 1.50)
	Mid-high	1,032	26.06	1.16	(0.98, 1.39)	2,305	17.53	1.07	(0.91, 1.26)	2,362	36.1	1.09	(0.91, 1.26)	2,304	13.8	1.01	(0.84, 1.22)
	High	1,080	22.41	ref		2,302	16.35	ref		2,352	33.02	ref		2,298	13.66	ref	
Education (30-64)	Low	1,177	11.27	2.02	(1.41, 2.90)	1,269	31.65	2.56	(1.68, 3.91)	1,269	48.07	1.82	(1.68, 3.91)	1,264	31.38	2.34	(1.50, 3.64)
	Middle	1,425	7.07	1.27	(0.88, 1.83)	3,296	16.17	1.31	(1.07, 1.60)	3,296	29.75	1.13	(1.07, 1.60)	3,293	15.69	1.17	(0.97, 1.41)
	High	281	5.57	ref		2,062	12.36	ref		2,062	26.41	ref		2,059	13.41	ref	
Education (≥65)	Low	1,995	45.67	1.07	(0.88, 1.30)	1,995	42.19	2.13	(1.41, 3.21)	1,996	68.44	1.41	(1.41, 3.21)	1,986	19.91	1.16	(0.79, 1.69)
	Middle	206	50.16	1.17	(0.91, 1.51)	206	40.44	2.04	(1.29, 3.22)	206	70.67	1.46	(1.29, 3.22)	205	16.05	0.93	(0.54, 1.59)
	High	234	42.73	ref		234	19.8	ref		234	48.51	ref		234	17.23	ref	
Occupation (30-64)	Physical work	791	18.37	3.26	(1.93, 5.50)	1,162	15.77	0.79	(0.58, 1.09)	1,162	26.83	0.78	(0.58, 1.09)	1,161	11.7	0.71	(0.53, 0.97)
	Service or sales	544	19.2	3.41	(1.99, 5.84)	1,183	19.02	0.96	(0.78, 1.18)	1,183	33.83	0.99	(0.78, 1.18)	1,180	15.37	0.94	(0.73, 1.20)
	Manager or office job	176	5.64	ref		1,801	19.87	Ref		1,801	34.32	ref		1,799	16.4	ref	

Notes: KNHANES = Korea National Health and Nutrition Examination Survey, EQ-5D = EuroQol five-dimension questionnaire, PR = Prevalence Ratios.

Table S3. Age-adjusted prevalences and prevalence ratios of 12 health conditions in women according to education, income and occupational groups in KNHANES 2010-2012. (continued)

		Suicidal ideation				Suicide attempts				Hepatitis B				Injury			
		N	Age standardized prevalence	PR *	95% CI	N	Age standardized prevalence	PR *	95% CI	N	Age standardized prevalence	PR *	95% CI	N	Age standardized prevalence	PR *	95% CI
Income	Low	2,035	27.62	2.21	(1.77, 2.75)	2,034	2.44	6.47	(2.17, 19.32)	1,874	2.89	0.67	(0.33, 1.35)	2,043	7.84	1.19	(0.80, 1.77)
	Mid-low	2,321	17.59	1.41	(1.16, 1.70)	2,321	0.73	1.95	(0.62, 6.09)	2,251	3.74	0.86	(0.60, 1.24)	2,323	5.32	0.81	(0.60, 1.08)
	Mid-high	2,304	15.32	1.22	(1.01, 1.49)	2,302	0.71	1.88	(0.60, 5.86)	2,250	3.67	0.85	(0.59, 1.22)	2,303	6.18	0.94	(0.71, 1.23)
	High	2,298	12.51	ref		2,298	0.38	ref		2,251	4.33	ref		2,301	6.6	ref	
Education (30-64)	Low	1,264	27.99	2.36	(1.49, 3.75)	1,263	0.7	2.98	(0.94, 9.41)	1,218	1.9	0.5	(0.27, 0.92)	1,269	8.55	1.49	(0.48, 4.61)
	Middle	3,293	14.29	1.2	(1.00, 1.45)	3,292	0.98	4.16	(1.62, 10.73)	3,179	4.02	1.06	(0.71, 1.58)	3,296	5.49	0.96	(0.70, 1.30)
	High	2,058	11.86	ref		2,058	0.23	ref		1,993	3.8	ref		2,062	5.74	ref	
Education (≥65)	Low	1,986	29.13	1.95	(1.25, 3.03)	1,986	0.77	1.62	(0.21, 12.55)	1,733	2.86	0.65	(0.21, 2.01)	1,996	8.41	1.33	(0.70, 2.55)
	Middle	205	13.01	0.87	(0.49, 1.53)	205	1.17	2.47	(0.21, 29.41)	182	1.13	0.26	(0.05, 1.36)	206	8.41	1.33	(0.58, 3.05)
	High	234	14.96	ref		234	0.47	ref		213	4.41	ref		234	6.31	ref	
Occupation (30-64)	Physical work	1,161	9.26	0.52	(0.40, 0.68)	1,161	0.28	0.52	(0.12, 2.29)	1,126	3.34	0.82	(0.45, 1.51)	1,162	7.32	1.23	(0.67, 2.26)
	Service or sales	1,179	16.8	0.95	(0.75, 1.21)	1,178	1.23	2.26	(0.65, 7.83)	1,147	3.95	0.97	(0.53, 1.78)	1,183	5.64	0.95	(0.61, 1.47)
	Manager or office job	1,799	17.65	ref		1,799	0.55	ref		1,725	4.06	ref		1,801	5.93	ref	

Notes: KNHANES = Korea National Health and Nutrition Examination Survey, EQ-5D = EuroQol five-dimension questionnaire, PR = Prevalence Ratios.

Table S4. Age-adjusted prevalences and prevalence differences of 12 health conditions in total participants according to education, income and occupational groups in KNHANES 2010-2012.

		Hypertension				Diabetes				Cancer				Ischemic heart disease			
		N	Age standardized prevalence	PD *	95% CI	N	Age standardized prevalence	PD *	95% CI	N	Age standardized prevalence	PD *	95% CI	N	Age standardized prevalence	PD *	95% CI
Income	Low	3,301	30.35	4.2	(0.92, 7.48)	2,867	12.65	3.79	(1.25, 6.33)	3,307	2.87	0.53	(-0.78, 1.84)	3,309	2.69	0.76	(-0.21, 1.73)
	Mid-low	4,033	27.72	1.57	(-0.77, 3.91)	3,767	9.18	0.32	(-1.19, 1.82)	4,050	2.24	-0.1	(-0.90, 0.71)	4,051	1.85	-0.08	(-0.80, 0.65)
	Mid-high	4,107	27.81	1.66	(-0.59, 3.91)	3,908	9.19	0.33	(-1.30, 1.95)	4,126	2.17	-0.17	(-0.93, 0.59)	4,127	2.11	0.18	(-0.61, 0.97)
	High	4,123	26.15	ref		3,961	8.86	ref		4,148	2.34	ref		4,148	1.93	ref	
Education (30-64)	Low	1,761	29.04	10.29	(1.70, 18.88)	1,664	12.46	6.83	(-0.78, 14.44)	1,768	1.67	0.24	(-0.99, 1.46)	1,769	1.4	0.49	(-0.33, 1.31)
	Middle	5,545	20.69	1.94	(-0.17, 4.05)	5,297	7.46	1.83	(0.44, 3.22)	5,577	1.77	0.34	(-0.30, 0.97)	5,577	0.97	0.05	(-0.46, 0.56)
	High	4,133	18.75	ref		3,969	5.63	ref		4,156	1.43	ref		4,157	0.91	ref	
Education (≥65)	Low	2,826	63.87	4.59	(-0.51, 9.69)	2,411	20.63	-0.71	(-5.00, 3.58)	2,833	4.5	-1.4	(-3.87, 1.07)	2,832	6.28	-1.15	(-3.90, 1.59)
	Middle	522	58.56	-0.72	(-7.16, 5.72)	458	24.54	3.2	(-2.84, 9.24)	522	6.08	0.18	(-3.19, 3.54)	522	8.52	1.09	(-2.35, 4.53)
	High	920	59.28	ref		835	21.34	ref		922	5.9	ref		922	7.44	ref	
Occupation (30-64)	Physical work	3,156	23.07	1.95	(-0.73, 4.63)	3,029	7.63	0.85	(-0.92, 2.61)	3,175	0.94	-0.1	(-0.64, 0.43)	3,176	1.01	-0.48	(-1.20, 0.24)
	Service or sales	1,723	21.08	-0.04	(-3.06, 2.98)	1,675	6.95	0.17	(-1.78, 2.11)	1,735	1.33	0.29	(-0.37, 0.96)	1,735	0.86	-0.63	(-1.45, 0.18)
	Manager or office job	2,877	21.12	ref		2,766	6.79	ref		2,895	1.04	ref		2,895	1.49	ref	

Notes: KNHANES = Korea National Health and Nutrition Examination Survey, EQ-5D = EuroQol five-dimension questionnaire, PR = Prevalence Ratios.

Table S4. Age-adjusted prevalences and prevalence differences of 12 health conditions in total participants according to education, income and occupational groups in KNHANES 2010-2012. (continued)

		Arthritis				Self-rated health				EQ-5D				Depressive mood			
		N	Age standardized prevalence	PD *	95% CI	N	Age standardized prevalence	PD *	95% CI	N	Age standardized prevalence	PD *	95% CI	N	Age standardized prevalence	PD *	95% CI
Income	Low	2,913	20.75	6.42	(3.78, 9.05)	3,312	30.01	16.41	(12.49, 20.33)	3,479	48.22	20.96	(16.87, 25.05)	3,291	22.39	11.41	(7.95, 14.87)
	Mid-low	2,376	16.91	2.58	(0.17, 4.99)	4,050	18.15	4.55	(2.64, 6.46)	4,204	33.25	5.99	(3.59, 8.39)	4,046	13.47	2.49	(0.62, 4.36)
	Mid-high	1,894	16.06	1.72	(-0.94, 4.38)	4,128	14.84	1.24	(-0.65, 3.13)	4,248	29.75	2.49	(0.20, 4.78)	4,130	10.57	-0.41	(-2.15, 1.33)
	High	2,032	14.33	ref		4,148	13.6	ref		4,264	27.26	ref		4,147	10.98	ref	
Education (30-64)	Low	1,634	17.25	11.15	(8.57, 13.72)	1,769	26.24	15.03	(5.82, 24.24)	1,769	41.63	21.47	(11.18, 31.76)	1,764	24.25	14.67	(4.48, 24.86)
	Middle	2,614	10.03	3.93	(1.62, 6.23)	5,577	14.44	3.23	(1.41, 5.05)	5,577	25.43	5.27	(3.15, 7.39)	5,572	13.12	3.54	(1.93, 5.15)
	High	778	6.1	ref		4,157	11.21	ref		4,157	20.16	ref		4,153	9.58	ref	
Education (≥65)	Low	2,832	35.58	20.37	(16.23, 24.51)	2,832	39.01	19.49	(15.44, 23.54)	2,833	64.2	25.39	(20.80, 29.98)	2,818	17.75	7.21	(4.26, 10.16)
	Middle	522	27.04	11.83	(6.13, 17.53)	522	30.58	11.06	(4.96, 17.16)	522	55.14	16.33	(9.63, 23.03)	519	12.7	2.16	(-1.95, 6.27)
	High	922	15.21	ref		922	19.52	ref		922	38.81	ref		920	10.54	ref	
Occupation (30-64)	Physical work	1,842	9.88	5.27	(3.02, 7.52)	2,989	11.67	-3.92	(-6.28, -1.56)	2,989	19.79	-7.79	(-10.66, -4.92)	2,987	9.11	-2.45	(-4.38, -0.52)
	Service or sales	784	12.45	7.84	(4.88, 10.81)	1,870	16.62	1.03	(-1.73, 3.79)	1,870	27.57	-0.01	(-3.01, 2.99)	1,867	12.64	1.08	(-1.26, 3.42)
	Manager or office job	653	4.61	ref		4,418	15.59	ref		4,419	27.58	ref		4,415	11.56	ref	

Notes: KNHANES = Korea National Health and Nutrition Examination Survey, EQ-5D = EuroQol five-dimension questionnaire, PR = Prevalence Ratios.

Table S4. Age-adjusted prevalences and prevalence differences of 12 health conditions in total participants according to education, income and occupational groups in KNHANES 2010-2012. (continued)

		Suicidal ideation				Suicide attempts				Hepatitis B				Injury			
		N	Age standardized prevalence	PD *	95% CI	N	Age standardized prevalence	PD *	95% CI	N	Age standardized prevalence	PD *	95% CI	N	Age standardized prevalence	PD *	95% CI
Income	Low	3,290	23.65	13.37	(10.07, 16.67)	3,289	2.34	1.98	(0.96, 3.00)	3,077	3.11	-1.7	(-3.30, -0.09)	3,300	7.57	0.98	(-1.14, 3.10)
	Mid-low	4,046	14.38	4.1	(2.24, 5.96)	4,046	0.67	0.31	(-0.08, 0.70)	3,955	3.97	-0.84	(-2.01, 0.33)	4,046	6.72	0.12	(-1.38, 1.63)
	Mid-high	4,130	11.77	1.49	(-0.33, 3.31)	4,128	0.44	0.08	(-0.27, 0.44)	4,069	4.24	-0.57	(-1.75, 0.62)	4,126	7.15	0.55	(-0.89, 1.99)
	High	4,146	10.28	ref		4,146	0.36	ref		4,104	4.8	ref		4,147	6.59	ref	
Education (30-64)	Low	1,764	22.15	12.76	(3.42, 22.11)	1,763	2.05	1.82	(0.27, 3.37)	1,699	2.13	-1.95	(-3.27, -0.64)	1,769	10.23	3.8	(-4.21, 11.81)
	Middle	5,572	12.29	2.9	(1.35, 4.45)	5,571	0.76	0.53	(0.17, 0.89)	5,414	4.09	0.01	(-1.06, 1.08)	5,577	7.11	0.68	(-0.70, 2.06)
	High	4,152	9.39	ref		4,152	0.23	ref		4,034	4.08	ref		4,157	6.43	ref	
Education (≥65)	Low	2,818	26.36	15.1	(11.73, 18.47)	2,818	0.97	0.56	(-0.06, 1.18)	2,484	1.54	-0.44	(-1.67, 0.80)	2,833	8.44	1.84	(-0.61, 4.29)
	Middle	518	14.96	3.7	(-0.96, 8.36)	518	1.48	1.07	(-0.08, 2.21)	471	1.34	-0.64	(-2.07, 0.79)	522	6.26	-0.34	(-3.52, 2.84)
	High	920	11.26	ref		920	0.41	ref		852	1.97	ref		922	6.6	ref	
Occupation (30-64)	Physical work	2,987	7.68	-5.04	(-6.86, -3.22)	2,987	0.17	-0.46	(-0.86, -0.05)	2,896	4.49	-0.31	(-1.77, 1.16)	2,989	6.25	-1.6	(-3.32, 0.12)
	Service or sales	1,866	13.71	0.99	(-1.45, 3.43)	1,865	1.03	0.41	(-0.45, 1.27)	1,820	4.4	-0.4	(-2.01, 1.22)	1,870	6.84	-1.02	(-2.90, 0.86)
	Manager or office job	4,414	12.72	ref		4,414	0.62	ref		4,274	4.8	ref		4,419	7.85	ref	

Notes: KNHANES = Korea National Health and Nutrition Examination Survey, EQ-5D = EuroQol five-dimension questionnaire, PD = Prevalence Difference.

Table S5. Age-adjusted prevalences and prevalence differences of 12 health conditions in men according to education, income and occupational groups in KNHANES 2010-2012.

		Hypertension				Diabetes				Cancer				Ischemic heart disease			
		N	Age standardized prevalence	PD *	95% CI	N	Age standardized prevalence	PD *	95% CI	N	Age standardized prevalence	PD *	95% CI	N	Age standardized prevalence	PD *	95% CI
Income	Low	1,259	33.2	3.27	(-2.25, 8.79)	1,101	14.1	2.17	(-1.66, 6.00)	1,260	1.6	-0.19	(-1.69, 1.30)	1,261	3.23	1.21	(-0.40, 2.81)
	Mid-low	1,714	31.12	1.19	(-2.50, 4.88)	1,608	9.88	-2.06	(-4.63, 0.52)	1,724	1.54	-0.25	(-1.39, 0.88)	1,724	1.86	-0.17	(-1.10, 0.77)
	Mid-high	1,811	30.71	0.78	(-2.80, 4.36)	1,726	10.55	-1.38	(-4.04, 1.28)	1,823	1.5	-0.3	(-1.38, 0.79)	1,823	2.48	0.46	(-0.62, 1.54)
	High	1,832	29.93	ref		1,772	11.93	ref		1,823	1.79	ref		1,846	2.03	ref	
Education (30-64)	Low	497	41.92	17.16	(-0.74, 35.06)	469	17.9	10.53	(-5.62, 26.68)	500	1.81	0.82	(-1.56, 3.20)	500	1.55	0.11	(-1.44, 1.65)
	Middle	2,266	28.13	3.37	(0.33, 6.41)	2,175	9.65	2.29	(0.37, 4.21)	2,281	0.71	-0.28	(-0.88, 0.32)	2,281	1.5	0.05	(-0.71, 0.82)
	High	2,078	24.76	ref		1,997	7.37	ref		2,095	0.99	ref		2,095	1.45	ref	
Education (≥65)	Low	835	53.52	-5.77	(-11.80, 0.26)	723	18.39	-6.52	(-11.60, -1.44)	837	5.31	0.18	(-2.78, 3.15)	837	6.79	-1.31	(-4.51, 1.90)
	Middle	316	57.85	-1.44	(-8.79, 5.91)	281	28.55	3.64	(-4.64, 11.92)	316	4.11	-1.02	(-4.53, 2.49)	316	9.38	1.28	(-3.09, 5.66)
	High	686	59.29	ref		624	24.91	ref		688	5.13	ref		688	8.1	ref	
Occupation (30-64)	Physical work	1,930	25.33	0.13	(-3.17, 3.43)	1,854	8.77	0.99	(-1.09, 3.08)	1,943	0.62	-0.13	(-0.67, 0.41)	1,943	1.03	-0.71	(-1.57, 0.16)
	Service or sales	631	25.88	0.68	(-3.71, 5.07)	621	9.94	2.16	(-0.72, 5.04)	638	0.32	-0.43	(-0.99, 0.14)	638	0.9	-0.84	(-1.86, 0.18)
	Manager or office job	1,736	25.2	ref		1,665	7.78	ref		1,750	0.75	ref		1,750	1.74	ref	

Notes: KNHANES = Korea National Health and Nutrition Examination Survey, EQ-5D = EuroQol five-dimension questionnaire, PD = Prevalence Difference.

Table S5. Age-adjusted prevalences and prevalence differences of 12 health conditions in men according to education, income and occupational groups in KNHANES 2010-2012. (continued)

		Arthritis				Self-rated health				EQ-5D				Depressive mood			
		N	Age standardized prevalence	PD *	95% CI	N	Age standardized prevalence	PD *	95% CI	N	Age standardized prevalence	PD *	95% CI	N	Age standardized prevalence	PD *	95% CI
Income	Low	1,097	7.15	1.23	(-1.37, 3.83)	1,262	28.48	18.1	(12.61, 23.59)	1,339	41.99	20.91	(15.10, 26.72)	1,255	16.84	8.53	(4.01, 13.05)
	Mid-low	1,033	7.22	1.3	(-1.06, 3.66)	1,724	15.71	5.33	(2.77, 7.89)	1,799	28.46	7.38	(3.99, 10.77)	1,725	9.38	1.07	(-1.31, 3.45)
	Mid-high	862	5.42	-0.51	(-2.91, 1.89)	1,823	12.13	1.75	(-0.66, 4.16)	1,886	23.34	2.26	(-1.03, 5.55)	1,826	7.36	-0.96	(-3.16, 1.24)
	High	952	5.92	ref		1,846	10.38	ref		1,912	21.08	ref		1,849	8.31	ref	
Education (30-64)	Low	457	2.52	1.05	(-0.02, 2.12)	500	15.69	4.97	(-0.47, 10.41)	500	29.18	12.86	(-2.34, 28.06)	500	9.71	2.43	(-2.35, 7.21)
	Middle	1,189	1.98	0.51	(-0.32, 1.34)	2,281	12.48	1.76	(-0.61, 4.13)	2,281	20.89	4.57	(1.63, 7.51)	2,279	10.27	2.99	(0.84, 5.14)
	High	497	1.47	ref		2,095	10.72	ref		2,095	16.32	ref		2,094	7.28	ref	
Education (≥65)	Low	837	13.16	4.68	(0.95, 8.40)	837	31.33	11.76	(6.56, 16.96)	837	53.92	18.06	(12.23, 23.89)	832	12.34	3.85	(0.24, 7.46)
	Middle	316	15.36	6.88	(1.61, 12.16)	316	24.64	5.07	(-1.98, 12.12)	316	45.8	9.94	(2.02, 17.86)	314	10.61	2.12	(-2.31, 6.55)
	High	688	8.48	ref		688	19.57	ref		688	35.86	ref		686	8.49	ref	
Occupation (30-64)	Physical work	1,051	4.81	0.69	(-1.81, 3.18)	1,827	10.55	-2.38	(-4.92, 0.16)	1,827	16.92	-6.92	(-10.27, -3.57)	1,826	7.74	-1.3	(-3.39, 0.79)
	Service or sales	240	2.71	-1.42	(-4.31, 1.48)	687	13.25	0.32	(-3.52, 4.16)	687	19.01	-4.83	(-9.17, -0.49)	687	8.76	-0.28	(-3.16, 2.60)
	Manager or office job	477	4.13	ref		2,618	12.93	ref		2,618	23.84	ref		2,616	9.04	ref	

Notes: KNHANES = Korea National Health and Nutrition Examination Survey, EQ-5D = EuroQol five-dimension questionnaire, PD = Prevalence Difference.

Table S5. Age-adjusted prevalences and prevalence differences of 12 health conditions in men according to education, income and occupational groups in KNHANES 2010-2012. (continued)

		Suicidal ideation				Suicide attempts				Hepatitis B				Injury			
		N	Age standardized prevalence	PD *	95% CI	N	Age standardized prevalence	PD *	95% CI	N	Age standardized prevalence	PD *	95% CI	N	Age standardized prevalence	PD *	95% CI
Income	Low	1,255	18.54	10.42	(6.14, 14.70)	1,255	2.32	1.96	(0.29, 3.62)	1,203	3.26	-1.96	(-4.20, 0.28)	1,257	7.46	1.03	(-2.04, 4.09)
	Mid-low	1,725	11	2.88	(0.52, 5.25)	1,725	0.6	0.24	(-0.30, 0.78)	1,704	4.21	-1.01	(-2.73, 0.70)	1,723	8.28	1.84	(-0.43, 4.11)
	Mid-high	1,826	8.04	-0.08	(-2.34, 2.19)	1,826	0.17	-0.19	(-0.64, 0.26)	1,819	4.76	-0.47	(-2.19, 1.26)	1,823	8.04	1.6	(-0.51, 3.71)
	High	1,848	8.12	ref		1,848	0.36	ref		1,853	5.22	ref		1,846	6.44	ref	
Education (30-64)	Low	500	10.51	2.99	(-1.38, 7.36)	500	3.85	3.66	(0.21, 7.10)	481	2.67	-2.2	(-4.87, 0.47)	500	13.42	6.55	(-8.08, 21.17)
	Middle	2,279	10.01	2.49	(0.38, 4.60)	2,279	0.51	0.31	(-0.18, 0.81)	2,235	5.27	0.4	(-1.27, 2.07)	2,281	9	2.12	(0.05, 4.20)
	High	2,094	7.52	ref		2,094	0.2	ref		2,041	4.87	ref		2,095	6.87	ref	
Education (≥65)	Low	832	19.51	9.28	(5.01, 13.55)	832	1.5	1.07	(0.03, 2.12)	751	1.64	-0.67	(-2.28, 0.94)	837	8.4	1.71	(-1.84, 5.27)
	Middle	313	16.19	5.96	(-0.22, 12.14)	313	1.66	1.24	(-0.15, 2.62)	289	2.29	-0.02	(-2.16, 2.12)	316	4.98	-1.7	(-5.40, 1.99)
	High	686	10.23	ref		686	0.43	ref		639	2.31	ref		688	6.69	ref	
Occupation (30-64)	Physical work	1,826	6.39	-3.94	(-5.94, -1.95)	1,826	0.12	-0.54	(-1.02, -0.07)	1,770	4.95	-0.27	(-2.07, 1.54)	1,827	6.24	-2.24	(-4.21, -0.28)
	Service or sales	687	9.43	-0.9	(-3.82, 2.02)	687	0.56	-0.1	(-0.93, 0.72)	673	4.89	-0.33	(-2.69, 2.03)	687	8.1	-0.38	(-3.47, 2.70)
	Manager or office job	2,615	10.33	ref		2,615	0.66	ref		2,549	5.22	ref		2,618	8.49	ref	

Notes: KNHANES = Korea National Health and Nutrition Examination Survey, EQ-5D = EuroQol five-dimension questionnaire, PD = Prevalence Difference.

Table S6. Age-adjusted prevalences and prevalence differences of 12 health conditions in women according to education, income and occupational groups in KNHANES 2010-2012.

		Hypertension				Diabetes				Cancer				Ischemic heart disease			
		N	Age standardized prevalence	PD *	95% CI	N	Age standardized prevalence	PD *	95% CI	N	Age standardized prevalence	PD *	95% CI	N	Age standardized prevalence	PD *	95% CI
Income	Low	2,042	27.11	5.66	(2.26, 9.06)	1,766	11.55	5.93	(2.78, 9.09)	2,047	4.15	1.02	(-1.22, 3.26)	2,048	2.27	0.68	(-0.33, 1.68)
	Mid-low	2,319	24.52	3.07	(0.48, 5.66)	2,159	8.64	3.03	(1.24, 4.81)	2,326	2.83	-0.29	(-1.44, 0.85)	2,327	1.85	0.26	(-0.68, 1.19)
	Mid-high	2,296	24.28	2.83	(0.26, 5.40)	2,182	7.74	2.13	(0.18, 4.08)	2,303	2.97	-0.15	(-1.32, 1.01)	2,304	1.75	0.16	(-0.85, 1.16)
	High	2,291	21.45	ref		2,189	5.61	ref		2,302	3.13	ref		2,302	1.6	ref	
Education (30-64)	Low	730	38.55	19.24	(7.73, 30.75)	1,195	10.69	7.31	(-0.13, 14.76)	1,268	1.41	-0.92	(-2.07, 0.23)	1,269	1.49	1.09	(0.43, 1.74)
	Middle	1,457	28.02	8.71	(3.36, 14.06)	3,122	5.52	2.15	(0.46, 3.84)	3,296	3.01	0.68	(-0.52, 1.88)	3,296	0.74	0.34	(-0.19, 0.87)
	High	749	19.31	ref		1,972	3.38	ref		2,061	2.33	ref		2,062	0.4	ref	
Education (≥65)	Low	1,445	85.13	-1.46	(-8.92, 6.00)	1,688	21.75	2.16	(-6.22, 10.54)	1,996	4.59	-2.49	(-6.47, 1.49)	1,995	6.35	-0.54	(-5.53, 4.44)
	Middle	144	88.35	1.76	(-7.43, 10.95)	177	18.88	-0.71	(-11.41, 9.98)	206	10.74	3.66	(-3.48, 10.81)	206	9.34	2.45	(-4.49, 9.38)
	High	155	86.59	ref		211	19.59	ref		234	7.08	ref		234	6.89	ref	
Occupation (30-64)	Physical work	1,226	17.27	5.74	(1.70, 9.78)	1,175	5.29	1.17	(-2.38, 4.73)	1,232	1.74	0.03	(-1.31, 1.37)	1,233	0.93	0.4	(-0.35, 1.15)
	Service or sales	1,092	16.85	5.32	(1.15, 9.49)	1,054	4.76	0.64	(-2.95, 4.23)	1,097	2.05	0.34	(-0.95, 1.63)	1,097	0.81	0.28	(-0.61, 1.17)
	Manager or office job	1,141	11.53	ref		1,101	4.12	ref		1,145	1.71	ref		1,145	0.53	ref	

Notes: KNHANES = Korea National Health and Nutrition Examination Survey, EQ-5D = EuroQol five-dimension questionnaire, PD = Prevalence Difference.

Table S6. Age-adjusted prevalences and prevalence differences of 12 health conditions in women according to education, income and occupational groups in KNHANES 2010-2012. (continued)

		Arthritis				Self-rated health				EQ-5D				Depressive mood			
		N	Age standardized prevalence	PD *	95% CI	N	Age standardized prevalence	PD *	95% CI	N	Age standardized prevalence	PD *	95% CI	N	Age standardized prevalence	PD *	95% CI
Income	Low	1,816	30.13	7.73	(3.59, 11.86)	2,050	30.76	14.41	(9.62, 19.20)	2,140	53.41	20.39	(15.15, 25.63)	2,036	26.96	13.3	(8.54, 18.06)
	Mid-low	1,343	25.18	2.77	(-1.07, 6.61)	2,326	20.29	3.94	(1.26, 6.62)	2,405	37.65	4.63	(1.42, 7.84)	2,321	17.2	3.54	(0.86, 6.22)
	Mid-high	1,032	26.06	3.65	(-0.52, 7.83)	2,305	17.53	1.18	(-1.54, 3.90)	2,362	36.1	3.08	(0.01, 6.15)	2,304	13.8	0.14	(-2.41, 2.69)
	High	1,080	22.41	ref		2,302	16.35	ref		2,352	33.02	ref		2,298	13.66	ref	
Education (30-64)	Low	1,177	11.27	5.7	(3.44, 7.95)	1,269	31.65	19.29	(6.86, 31.72)	1,269	48.07	21.66	(8.13, 35.19)	1,264	31.38	17.97	(4.85, 31.09)
	Middle	1,425	7.07	1.5	(-0.62, 3.63)	3,296	16.17	3.81	(1.12, 6.50)	3,296	29.75	3.34	(0.15, 6.53)	3,293	15.69	2.28	(-0.36, 4.92)
	High	281	5.57	ref		2,062	12.36	ref		2,062	26.41	ref		2,059	13.41	ref	
Education (≥65)	Low	1,995	45.67	2.94	(-5.54, 11.42)	1,995	42.19	22.39	(13.98, 30.80)	1,996	68.44	19.93	(11.10, 28.76)	1,986	19.91	2.68	(-3.95, 9.31)
	Middle	206	50.16	7.43	(-4.32, 19.18)	206	40.44	20.64	(8.97, 32.31)	206	70.67	22.16	(11.26, 33.06)	205	16.05	-1.18	(-10.10, 7.74)
	High	234	42.73	ref		234	19.8	ref		234	48.51	ref		234	17.23	ref	
Occupation (30-64)	Physical work	791	18.37	12.74	(8.93, 16.54)	1,162	15.77	-4.1	(-9.40, 1.20)	1,162	26.83	-7.49	(-13.40, -1.58)	1,161	11.7	-4.7	(-8.78, -0.62)
	Service or sales	544	19.2	13.57	(8.95, 18.18)	1,183	19.02	-0.85	(-4.86, 3.16)	1,183	33.83	-0.49	(-5.22, 4.24)	1,180	15.37	-1.03	(-4.96, 2.90)
	Manager or office job	176	5.64	ref		1,800	19.87	ref		1,801	34.32	ref		1,799	16.4	ref	

Notes: KNHANES = Korea National Health and Nutrition Examination Survey, EQ-5D = EuroQol five-dimension questionnaire, PD = Prevalence Difference.

Table S6. Age-adjusted prevalences and prevalence differences of 12 health conditions in women according to education, income and occupational groups in KNHANES 2010-2012. (continued)

		Suicidal ideation				Suicide attempts				Hepatitis B				Injury			
		N	Age standardized prevalence	PD *	95% CI	N	Age standardized prevalence	PD *	95% CI	N	Age standardized prevalence	PD *	95% CI	N	Age standardized prevalence	PD *	95% CI
Income	Low	2,035	27.62	15.11	(10.40, 19.82)	2,034	2.44	2.06	(0.72, 3.40)	1,874	2.89	-1.44	(-3.64, 0.76)	2,043	7.84	1.24	(-1.78, 4.26)
	Mid-low	2,321	17.59	5.08	(2.36, 7.80)	2,321	0.73	0.36	(-0.22, 0.94)	2,251	3.74	-0.59	(-2.06, 0.89)	2,323	5.32	-1.28	(-3.03, 0.47)
	Mid-high	2,304	15.32	2.81	(0.16, 5.46)	2,302	0.71	0.33	(-0.24, 0.90)	2,250	3.67	-0.66	(-2.12, 0.80)	2,303	6.18	-0.42	(-2.19, 1.34)
	High	2,298	12.51	ref		2,298	0.38	ref		2,251	4.33	ref		2,301	6.6	ref	
Education (30-64)	Low	1,264	27.99	16.13	(3.75, 28.51)	1,263	0.7	0.47	(-0.13, 1.06)	1,218	1.9	-1.9	(-3.48, -0.31)	1,269	8.55	2.8	(-6.73, 12.34)
	Middle	3,293	14.29	2.43	(0.10, 4.76)	3,292	0.98	0.74	(0.24, 1.25)	3,179	4.02	0.22	(-1.33, 1.77)	3,296	5.49	-0.26	(-1.99, 1.48)
	High	2,058	11.86	ref		2,058	0.23	ref		1,993	3.8	ref		2,062	5.74	ref	
Education (≥65)	Low	1,986	29.13	14.17	(7.19, 21.15)	1,986	0.77	0.29	(-0.73, 1.32)	1,733	2.86	-1.55	(-6.42, 3.33)	1,996	8.41	2.1	(-2.11, 6.30)
	Middle	205	13.01	-1.95	(-9.99, 6.09)	205	1.17	0.69	(-1.30, 2.69)	182	1.13	-3.28	(-8.27, 1.72)	206	8.41	2.1	(-3.94, 8.13)
	High	234	14.96	ref		234	0.47	ref		213	4.41	ref		234	6.31	ref	
Occupation (30-64)	Physical work	1,161	9.26	-8.39	(-11.87, -4.91)	1,161	0.28	-0.26	(-0.86, 0.34)	1,126	3.34	-0.72	(-2.98, 1.55)	1,162	7.32	1.39	(-2.83, 5.60)
	Service or sales	1,179	16.8	-0.85	(-5.01, 3.31)	1,178	1.23	0.69	(-0.47, 1.84)	1,147	3.95	-0.11	(-2.53, 2.30)	1,183	5.64	-0.29	(-2.82, 2.24)
	Manager or office job	1,799	17.65	ref		1,799	0.55	ref		1,725	4.06	ref		1,801	5.93	ref	

Notes: KNHANES = Korea National Health and Nutrition Examination Survey, EQ-5D = EuroQol five-dimension questionnaire, PD = Prevalence Difference.
